# Quantitative analysis of retinal layers' optical intensities on 3D optical coherence tomography for central retinal artery occlusion

**DOI:** 10.1038/srep09269

**Published:** 2015-03-18

**Authors:** Haoyu Chen, Xinjian Chen, Zhiqiao Qiu, Dehui Xiang, Weiqi Chen, Fei Shi, Jianlong Zheng, Weifang Zhu, Milan Sonka

**Affiliations:** 1 Joint Shantou International Eye Center, Shantou University and the Chinese University of Hong Kong, Shantou, China; 2 School of Electronics and Information Engineering, Soochow University, Suzhou, China; 3 Iowa Institute for Biomedical Imaging and Department of Electrical and Computer Engineering, University of Iowa, Iowa, IA 52242, USA

## Abstract

Optical coherence tomography (OCT) provides not only morphological information but also information about layer-specific optical intensities, which may represent the underlying tissue properties. The purpose of this study is to quantitatively investigate the optical intensity of each retinal layers in central retinal artery occlusion (CRAO). Twenty-nine CRAO cases at acute phase and 33 normal controls were included. Macula-centered 3D OCT images were segmented with a fully-automated Iowa Reference Algorithm into 10 layers. Layer-specific mean intensities were determined and compared between the patient and control groups using multiple regression analysis while adjusting for age and optical intensity of the entire region. The optical intensities were higher in CRAO than in controls in layers spanning from the retinal ganglion cell layer to outer plexiform layer (standardized beta = 0.657 to 0.777, all p < 0.001), possibly due to ischemia. Optical intensities were lower at the photoreceptor, retinal pigment epithelium (RPE), and choroid layers (standardized beta = −0.412 to −0.611, all p < 0.01), possibly due to shadowing effects. Among the intraretinal layers, the inner nuclear layer was identified as the best indicator of CRAO. Our study provides *in vivo* information of the optical intensity changes in each retinal layer in CRAO patients.

Central retinal artery occlusion (CRAO) is an ocular emergency that can lead to severe ischemia of the retina and cause a sudden loss of vision^1^. In the acute phase, whitish opacification of the retina is present at the posterior pole except fovea, which does not have inner retinal layers. The opacification typically resolves spontaneously within a month^2^. On fundus fluorescein angiography, delay of arterio-venous transit time and retinal artery filling time is usually observed^3^. However, fluorescein angiography is invasive, and subject to severe complications, including a possibility of allergic shock. Furthermore, fluorescein angiography provides only limited information about the severity of retinal damage^4^.

Optical coherence tomography (OCT) is an *in vivo*, non-invasive technology providing cross-sectional images of the retina and allowing quantitative measurements of retinal thickness^5^. In 2006, it was first reported that the retinal thickness was increased in the acute phase of CRAO, and was subsequently reduced at follow-up of several months. However, the observed extent of retinal edema did not correlate with the visual prognosis^6^. Later, with the introduction of spectral domain OCT with higher resolution, it was found that the thickness of inner retina decreased in chronic phase of CRAO but there was no similar change in the outer retina^7^. Recently, it was reported that in the acute phase of branch retinal artery occlusion (BRAO), the thicknesses were markedly increased in the inner layers including the retinal nerve fiber layer (RNFL)/ganglion cell layer (GCL), inner plexiform layer (IPL) and inner nuclear layer (INL)/outer plexiform layer (OPL), and mildly increased in the outer nuclear layer (ONL). In the chronic phase, reduction of thickness was noted with an observed loss of differentiation between the IPL and INL/OPL. In contrast, no thickness change of the photoreceptor/retinal pigment epithelium (RPE) layer was observed in either the acute or chronic phase^8^.

Besides change of the retinal thickness, it was also observed that the reflectivity increased in the inner retina and correspondingly decreased in the outer retina in the acute phase of CRAO. In the chronic phase, the increased reflectivity of the inner retinal layers were reduced and the decreased reflectivity in the outer retina layer recovered^9^,^10^. However, the observed reflectivity changes have not been assessed quantitatively in any of the previous studies.

OCT is not only valuable in providing morphological images, but it also offers quantitative measurements of local optical intensities (also called optical density or reflectivity) of the underlying normal and/or pathological tissues. For example, it was previously reported that the optical intensities of the intraretinal or subretinal fluids are different in various diseases^11^,^12^ and correlate with visual function in diabetic macular edema^13^. Similarly, optical intensity of pigment epithelial detachment can be used to differentiate serous, fibrovascular and drusenoid types^14^. Retinal layers' intensities show a better performance in discriminating mild diabetic retinopathy and control eyes than retinal layer thickness values^15^. In glaucoma, the reflectance of the retinal nerve fiber layer decreases compared to that of normal controls^16^, and reflectance changes can be seen even before any thinning is detected^17^. These results suggested that the optical intensities of intraretinal or subretinal spaces can be used as biomarkers and provide clues to the pathogenesis of retinal diseases.

In this study, we quantitatively investigated the reflectivity, or optical intensity in each of 10 retinal layers identified in 3D-OCT images from patients with CRAO using a validated automatic computer algorithm and compared with the same measurements obtained from normal controls^18^,^19^,^20^,^21^,^22^.

## Results

**Figure 1:**
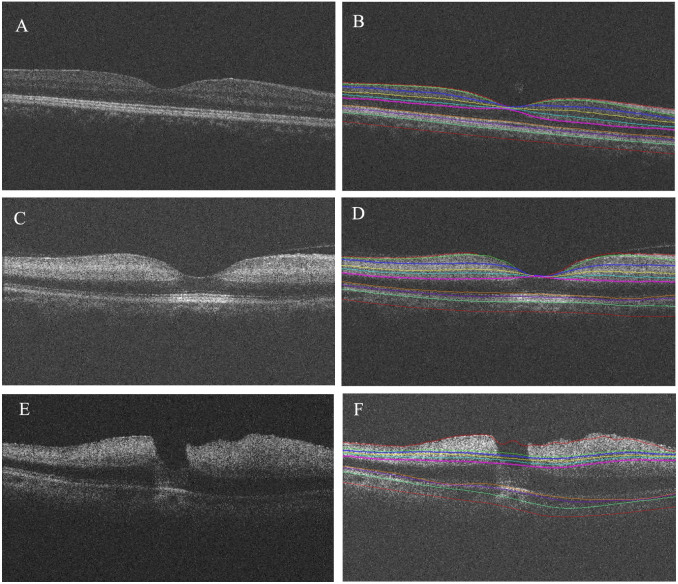
Segmented surfaces and regions on macular spectral domain optical coherence tomography. (A.B.): control subjects; (C.D.): central retinal artery occlusion patients with correctly segmented retinal layers; (E.F.): central retinal artery occlusion patients with substantially increased brightness of the inner retina and related insufficient separation of inner retinal layers on OCT, causing layer segmentation errors – an example is shown. (A.C.E): original OCT images; (B.D.F): segmentation results.

Figure 1 shows examples of our OCT image segmentation. No segmentation error was observed in any of the 33control subjects (Figure 1A.B.). Correct 3D OCT segmentation was achieved in 29 of 40 CRAO patients (Figure 1C.D.). The 29 images were included in further analysis. The segmentation of the OCT images of the remaining 11 CRAO cases (Figure 1E.F.) was not good due to insufficient quality of OCT image data. These cases were not included in further analysis. In these cases, the optical intensity was very high at each layer of inner retina and the overall brightness (saturation) of image intensities made finding interfaces between layers impossible.

Due to the difficulty to enroll exactly age-matched normal controls, the CRAO subjects were slightly older compared to the control group (77.0 ± 5.7 vs 71.9 ± 4.5 years old, p < 0.001). There was no statistically significant difference of gender distribution or perceived quality of included images between the CRAO and control groups (Table 1). No statistically significant correlation was found between the optical intensities of retinal layers and the disease duration from the onset to the image acquisition (all p > 0.05).

**Figure 2:**
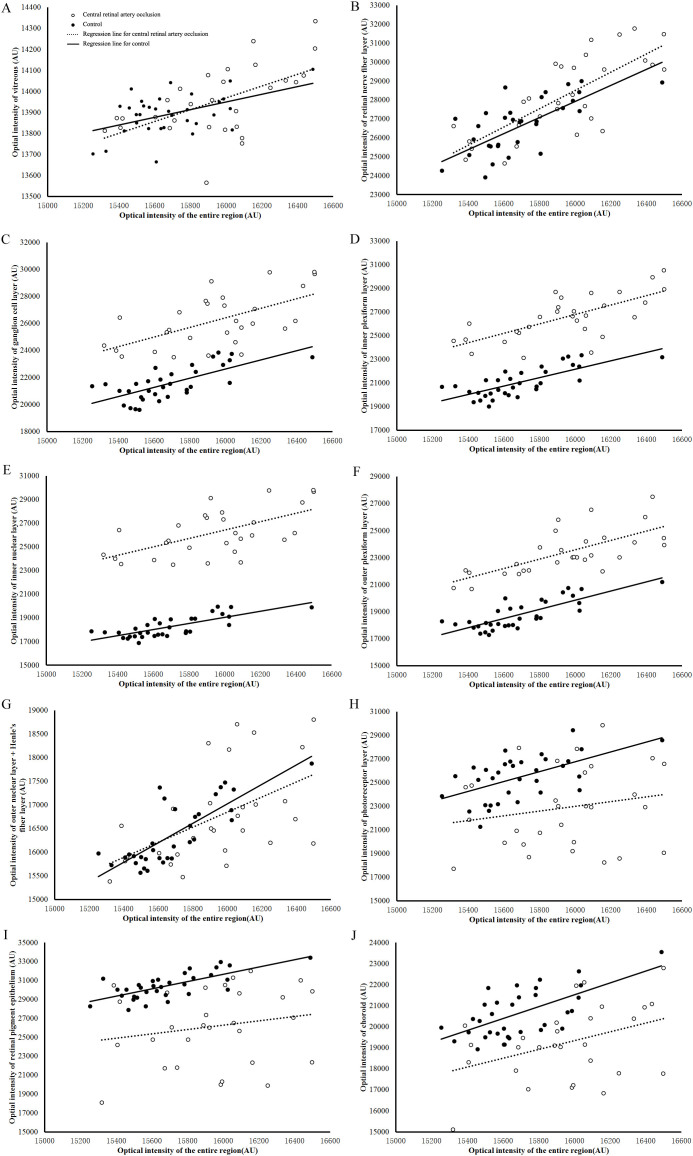
Regression of optical intensities in each layer with intensities of the entire retinal region in retinal artery occlusion patients and controls. Circles represent patients with central retinal artery occlusion, black dots represent control subjects.

The mean and standard deviation of optical intensities and optical intensity ratios in each layer are shown in Table 2. Results of multi-linear regression after adjusting for optical intensity of the entire region and age are given in Table 2 and Figure 2. There was no statistically significant difference of optical intensities between the CRAO and control subjects in the vitreous and RNFL (standardized beta = 0.160 and 0.050 respectively, both p > 0.5, Table 2 and Figure 2A.B.). Optical intensities in GCL, IPL, INL and OPL were higher in CRAO compared to controls (standardized beta = 0.657, 0.702, 0.777 and 0.694, respectively, all p < 0.001, Table 2 and Figure 2 C.D.E.F). Optical intensity at ONL + HFL was not different between the CRAO and control groups (standardized beta = 0.047, p > 0.5, Table 2 and Figure 2 G.). Optical intensities at the photoreceptor, RPE, and choroidal layers were lower in the CRAO cases compared to controls (standardized beta = −0.412, −0.611 and −0.559, all p < 0.001, Table 2 and Figure 2 H.I.J.). Discriminant analysis found that the optical intensity of INL was most strongly associated with the CRAO disease status (Wilks' Lambda = 0.641).

## Discussion

In this study, we quantitatively investigated the optical intensities in each segmented retinal layer detected from 3D-OCT for CRAO patients and normal controls. The results showed that the optical intensities of vitreous, RNFL and ONL were not different between CRAO and controls. The optical intensities of layers in the inner retina, from GCL to OPL, were higher in CRAO than in controls, with the most prominent increase identified at the INL. While the optical intensities from the photoreceptor to choroidal layers were lower in CRAO patients than in controls, the photoreceptor layer exhibited the most pronounced OCT image intensity decrease.

It is well known that there are two sources of blood supply to the neurosensory retina; the central retinal artery and the choroidal blood vessels, which supply the inner and outer retina, respectively. There are two layers of capillary networks originating from the branches of the central retinal artery. The inner capillary network lies within the GCL. The outer capillary network runs from the IPL to the OPL through the INL^23^. Our results showed that the optical intensities increased from the GCL to the OPL in CRAO patients. This finding shows a locational correspondence of image intensity increases with layers supplied by the central retinal artery. On histology of a CRAO mouse model, pyknotic nuclei, vacuolated spaces, and degenerative changes were noted in the GCL and INL^24^. In this study, the maximum increases of optical intensity or optical intensity ratio were detected in the INL. Furthermore, discriminant analysis found that the optical intensity of INL is the best indicator of CRAO. Recently, it was reported that deep capillary ischemia frequently manifests itself by increased optical intensities in the middle retinal layers, especially the INL^25^. There is a large number of metabolically active cells in the INL and the INL is surrounded by a deep capillary network branched from the central retinal artery system and is therefore subject to ischemia.

To our surprise, the optical intensity of RNFL was not different between the CRAO and control groups. The blood supply of RNFL is also provided from the central retinal artery system. In a pathological study of human autopsy eyes with CRAO, severe edema of the RNFL was frequently noted^26^. It is conceivable that edema of RNFL does not affect its OCT optical intensity. The exact cellular and molecular mechanism of change of optical intensity in CRAO remains unknown and deserves further investigation.

The ONL, photoreceptor, RPE and choroidal layers are not supplied by the retinal artery and its branches. Animal and human autopsy studies previously showed that the outer retina does not change in CRAO^24^,^26^. Our results found that the optical intensities in the outer retina from the photoreceptor layer to the choroid were reduced in the acute CRAO phase, especially in the photoreceptor and RPE layers. However, the optical intensities of the photoreceptor and RPE layers are still high at the foveal region, where no layered inner retinal structure is present (Figure 1 C.D.E.F.) On ophthalmoscopic examination, the whitish opacification of the CRAO retina is caused by reduced transparence of the inner retina, and the cherry-red spot at the fovea is due to a relative transparency of the fovea devoid of the inner retina layer tissue^27^. This evidence suggests that the reduction of optical intensities in outer layers may be associated with a shadowing effect caused by increased optical density in the inner retina as observed on CRAO patients.

This study used a validated automatic segmentation method^20^,^21^,^28^ to quantify the optical intensities in each retinal layer on 3D OCT for CRAO patients and normal controls. With the advantage of this method, changes of optical intensity in each layer can be quantified and objectively compared with normal controls. As a result, the optical intensity changes in the INL layer were identified as being of the highest significance for discriminating patients suffering from CRAO from normal subjects. The quantitative values of optical intensity may coincide with the severity of ischemia and may thus be used for *in vivo* future studies of OCT-derived disease severity.

Increased optical intensity in the inner retina was also observed in other disorders with retinal ischemia, such as diabetic retinopathy, retinal vein occlusion, hypertensive retinopathy, radiation retinopathy, HIV retinopathy^29^, Purtscher's retinopathy, and Purtscher-like retinopathy^30^,^31^. Our method could also be applied in these diseases to quantify the severity of ischemia and future studies should answer questions about the utility of our approach in this group of retinal diseases.

We recognize some limitations of the presented study. First, the segmentation of the OCT images of the remaining 11 CRAO cases (Figure 1E.F.) was not good due to insufficient quality of OCT image data. Therefore these cases were not included in further analysis. In these cases, the optical intensity was very high at each layer of inner retina and the overall brightness (saturation) of image intensities made finding interfaces between layers impossible. Second, although we used a validated algorithm for segmentation and the segmentation results were reviewed by a retinal specialist, we cannot rule out small segmentation errors of 1–2 pixels, which may have affected the results, especially for some extremely thin intraretinal layers. Third, we did not detect and exclude the retinal blood vessels and their shadows in analysis. The shadowing effect of retinal blood vessels on OCT may reduce the optical intensity of the structure beneath the vessel and affect the results, although it only accounts for a small portion of the analyzed OCT image data and thus has a minimal effect on the presented results. Fourth, the algorithm cannot distinguish the NFL from the ILM. This limitation may affect the results, especially for the RNFL temporal to the foveal center, which is very thin. Fifth, the surface between the ONL and HFL cannot be identified with the current algorithm. Therefore, the result of no difference of optical intensity in ONL + HFL may not show the possible intensity changes in these two individual layers when considered separately.

In conclusion, the OCT optical intensity of inner retina increases in patients with CRAO compared to normal controls, possibly due to layer-specific ischemia, while the optical intensities of the outer retina and the choroid decrease, possibly due to a shadowing effect associated with the inner retinal density increases.

## Methods

### Study subjects

This study was approved by the Institutional Review Board of the Joint Shantou International Eye Center (JSIEC), Shantou University and the Chinese University of Hong Kong and adhered to the tenets of the Declaration of Helsinki. Because of its retrospective nature, an informed consent was not required from subjects. The medical records and OCT database of JSIEC from January 2008 to June 2013 were searched and reviewed. Forty eyes of 40 patients diagnosed as CRAO who received 3D-OCT examination within one week of onset were included. Since most CRAO patients are elderly, we included 33 eyes of 33 subjects aged > = 65 years old without any retinal disorder or high myopia were included as controls. All the study subjects received comprehensive ophthalmic examinations including fundus photography and spectral domain 3D-OCT examination.

### Optical coherence tomography

Spectral domain OCT examination was performed using Topcon 3D OCT-1000 (Topcon Corporation, Tokyo, Japan). Macula was scanned using standard 6 × 6 mm protocol, in which 3D acquisition consisted of 64 B-scan slices. Fundus photographs were obtained from each subject at the same time. The OCT image size was 512 × 64 × 480 voxel. Image quality index was provided by the on-board OCT software. The raw images were exported from the OCT machine in “.fds” format for analysis.

### Image analysis

Ten surfaces were automatically segmented using the Iowa Reference Algorithm^18^,^19^,^20^,^21^,^22^. The following layers were obtained between the surfaces, vitreous, NFL, GCL, IPL, INL, OPL, ONL + Henle's fiber layer (HFL), photoreceptor and RPE. The surface between the choroid and the sclera is difficult to identify for Topcon 3D-1000 images, therefore the region, 25 pixels (about 125 μm) wide, immediately under the RPE was used to represent the choroid. The segmentation results were reviewed by a retinal specialist (H.C.) and the patients' image with segmentation error were excluded from further analysis. Layer-specific mean intensities were calculated for each layer and for each individual. Interpreting the raw scanned data as 16-bit gray-scale images resulted in 65,536 levels of gray, ranging from 0 to 65,535. Because raw images were used, intensity was expressed in arbitrary units (AU).

### Statistical analysis

The means and standard deviations of optical intensities in all subjects were calculated for all layers. The relationship between the duration from the disease onset to image acquisition and the determined optical intensities of retinal layers were assessed using Pearson's correlation. The age and image quality were compared between the CRAO patients and the normal controls by Student's independent *t* test. The male to female gender ratio between the two groups were compared using chi-square test. Our previous study found that the retinal layers' OCT optical intensity are highly correlated with the OCT optical intensity of the entire scanned region^32^. Therefore, the optical intensities in each layer on OCT were compared between CRAO and control subjects using multiple linear regression analysis adjusted for optical intensity of the entire scanned region and for age. The ratios of retinal layer optical intensities divided by that of the entire scanned region were calculated. Discriminant analysis was conducted to investigate which layer is the best indicator of CRAO. SPSS statistical software (version 20.0, IBM Corp. Armonk, NY) was used to conduct the statistical analyses. Sigmaplot (version 12.5, Systat Inc. Chicago, IL) and Excel (version 2013, Microsoft, Corp. Redmond, WA) were used to draw plots.

## Author Contributions

H.C. and X.C. designed the study. Z.Q., W.C. and J.Z. collect patients' information. X.C., D.X., F.S. and W.Z. analyze OCT images. H.C. and X.C. conducted statistical analysis. H.C. wrote the main manuscript text. X.C. and M.S. revised themanuscript.

## Figures and Tables

**Table 1:** Comparison of demographic and image quality information between normal subjects and retinal artery occlusion

	Normal	CRAO	P value
N	33	29	
Age (years)	71.9 ± 4.5	77.0 ± 5.7	<0.001
Gender (F/M)	19/14	14/15	0.611
Image quality	47.8 ± 6.4	50.9 ± 11.1	0.354

**Table 2:** Comparison of optical intensity in each layersbetween central retinal artery occlusion and control adjusting for age and the optical intensity of the entire scanned regions

	Optical intensity		Optical intensity ratio		Unstandardized beta	Standardized beta	p
	Control	CRAO	Control	CRAO			
Vitreous	13893.9 ± 96.5	13953.9 ± 164.6	0.89 ± 0.01	0.88 ± 0.02	4.2	0.160	0.901
Retinal nerve fiber layer	26598.5 ± 1598.1	28232.6 ± 2120.0	1.69 ± 0.09	1.77 ± 0.11	202.6	0.050	0.584
Retinal ganglion cell layer	21567.8 ± 1301.4	26219.3 ± 1980.1	1.37 ± 0.06	1.64 ± 0.11	3709.4	0.657	<0.001
Inner plexiform layer	21052.8 ± 1211.5	26559.5 ± 1915.7	1.34 ± 0.06	1.66 ± 0.10	4441.0	0.702	<0.001
Inner nuclear layer	18235.6 ± 857.9	23899.1 ± 1758.6	1.16 ± 0.04	1.50 ± 0.09	4863.3	0.777	<0.001
Outer plexiform layer	18791.6 ± 1084.7	23389.7 ± 1660.4	1.15 ± 0.03	1.40 ± 0.10	3708.8	0.694	<0.001
Outer nuclear layer + Henle's fiber layer	16380.9 ± 657.5	16757.9 ± 1002.9	1.04 ± 0.03	1.05 ± 0.05	80.3	0.047	0.664
Photoreceptor	25458.3 ± 1893.3	22885.4 ± 3406.9	1.62 ± 0.11	1.44 ± 0.21	−2440.8	−0.412	0.001
Retinal pigment epithelium	30454.5 ± 1375.2	26160.6 ± 3998.0	1.94 ± 0.07	1.64 ± 0.25	−4376.7	−0.611	<0.001
Choroid	20638.7 ± 1130.9	19224.1 ± 1753.5	1.32 ± 0.06	1.21 ± 0.10	−1787.7	−0.559	<0.001
Entire region	15688.0 ± 252.7	15943.2 ± 333.4	NA	NA	NA	NA	NA

NA: not applicable; CRAO: central retinal artery occlusion.
